# A Distinct Peripheral Blood Monocyte Phenotype Is Associated with Parasite Inhibitory Activity in Acute Uncomplicated *Plasmodium falciparum* Malaria

**DOI:** 10.1371/journal.ppat.1000631

**Published:** 2009-10-23

**Authors:** Pattamawan Chimma, Christian Roussilhon, Panudda Sratongno, Ronnatrai Ruangveerayuth, Kovit Pattanapanyasat, Jean-Louis Pérignon, David J. Roberts, Pierre Druilhe

**Affiliations:** 1 Bio-medical Parasitology Unit, Institut Pasteur, Paris, France; 2 Center of Excellence for Flow Cytometry, Office for Research and Development, Faculty of Medicine, Siriraj Hospital, Mahidol University, Bangkok, Thailand; 3 Mae Sot Hospital, Mae Sot, Tak, Thailand; 4 Nuffield Department of Clinical Laboratory Sciences, Oxford, United Kingdom; 5 National Blood Service Oxford Centre, John Radcliffe Hospital, Oxford, United Kingdom; Case Western Reserve University, United States of America

## Abstract

Monocyte (MO) subpopulations display distinct phenotypes and functions which can drastically change during inflammatory states. We hypothesized that discrete MO subpopulations are induced during malaria infection and associated with anti-parasitic activity. We characterized the phenotype of blood MO from healthy malaria-exposed individuals and that of patients with acute uncomplicated malaria by flow cytometry. In addition, MO defense function was evaluated by an *in vitro* antibody dependent cellular inhibition (ADCI) assay. At the time of admission, the percentages and absolute numbers of CD16^+^ MO, and CCR2^+^CX3CR1^+^ MO, were high in a majority of patients. Remarkably, expression of CCR2 and CX3CR1 on the CD14^high (hi)^ MO subset defined two subgroups of patients that also differed significantly in their functional ability to limit the parasite growth, through the ADCI mechanism. In the group of patients with the highest percentages and absolute numbers of CD14^hi^CCR2^+^CX3CR1^+^ MO and the highest mean levels of ADCI activity, blood parasitemias were lower (0.14±0.34%) than in the second group (1.30±3.34%; *p* = 0.0053). Data showed that, during a malaria attack, some patients' MO can exert a strong ADCI activity. These results bring new insight into the complex relationships between the phenotype and the functional activity of blood MO from patients and healthy malaria-exposed individuals and suggest discrete MO subpopulations are induced during malaria infection and are associated with anti-parasitic activity.

## Author Summary

Blood monocytes (MO) belong to the first line of defense against infectious pathogens, but little is known of MO phenotype and function during acute malaria infection. In patients with acute uncomplicated malaria, we identified a unique, and so far not described, minor population of MO. The percentage and absolute number of MO with this unusual phenotype was high in patients with low level of blood parasitemia, suggesting that activation of this particular subset of blood MO was potentially associated with the control of parasite infection. Indeed, the MO from these patients were the most efficient in the assay of antibody- and cellular-dependent inhibition of parasite multiplication (ADCI). These results support the hypothesis that discrete MO subpopulations are induced during malaria infection and are associated with anti-parasitic activity.

## Introduction

Innate defenses against malaria play a vital role in the clearance of *Plasmodium*-infected red blood cells (iRBCs) in murine and human infections [1]–[5]. This innate response against iRBCs is, at least in part, related to the functional activity of monocytes (MO) - macrophages and/or polymorphonuclear leukocytes. These myeloid cells can also modulate the inflammatory process and trigger the adaptive immune responses. Circulating blood MO, not only contribute directly to defense against *Plasmodium* parasites by their phagocytic activity, but also achieve parasite killing through an indirect mechanism known as antibody-dependant cellular inhibition (ADCI) [6]. Moreover, MO also supply peripheral tissues with macrophage and dendritic cell (DC) precursors.

The generic name ‘monocyte’ corresponds to a very large number of distinct phenotypes representing a highly heterogeneous population of cells, which is reflected in the complex MO response to *falciparum* malaria. The existence of distinct MO populations in human blood was initially described by Ziegler-Heitbrock and it is clearly established that subsets of MO can be influenced by infection [7]–[9]. Human MO can be divided into 2 major subsets by the differential expression of lipopolysaccharide (LPS) receptor (CD14) and the low affinity Fcγ receptor III (CD16). These two MO subsets vary in chemokine receptor, adhesion molecule expression, migratory and differentiation properties [9]–[11]. Up to 90–95% of the blood MO are CD14^hi^ CD16^−^, they are usually known as “classical” or “resting” MO, and express CCR2, CD64, CD62L. The best documented function of “classical” MO is the removal and recycling of apoptotic neutrophils at sites of inflammation [12]. It has recently been reported that CD16^+^ MO present different levels of CD14 expression [13] and correspond to two sub-groups of cells. The CD14^dim^ CD16^+^ secrete Tumor Necrosis Factor-α (TNF-α) [14] and correspond to “pro-inflammatory” MO, whereas the CD14^hi^ CD16^+^, sometimes called “intermediate” MO, exhibit intense HLA-DR expression [15] and, as main producers of IL-10, might represent an “anti-inflammatory” component of the MO subsets induced in response to infectious pathogens. The two CD16^+^ MO subsets greatly expand in various infectious and inflammatory diseases and differ not only in phenotype but also in function. An increase in the proportion of CD16^+^ MO expressing higher CCR5 levels than CD14^hi^ CD16^−^ MO has been reported in *P. falciparum* infected pregnant women [16] and a potential role for CD16^+^ MO in the pathogenesis of maternal malaria has also been suspected [16],[17]. Nevertheless, the precise pathophysiological role of this CD16^+^ CCR5^+^ MO subset remains unclear [18] and the functional significance of changes in MO phenotypes in different presentations of clinical malaria is unclear [19].

As in all infectious and inflammatory situations, the balance between the pro- and anti-inflammatory MO subsets is strongly regulated and this balance may influence the parasitological and clinical outcomes of human malaria episodes, so that characterizing changes occurring in the MO subsets and functional phenotype during acute malaria infection would allow an assessment of the contribution of diverse cell populations to infection and the inflammatory response [20]. The Triggering Receptor Expressed on Myeloid Cells–1 (TREM-1) is a marker of activated MO. This transmembrane receptor was recently implicated as having a critical role in regulating the function of activated neutrophils and MO/ macrophages in both innate and adaptive immunity [21]. TREM-1 is able to enhance the secretion of pro-inflammatory cytokines during acute inflammation and/or bacterial or fungal infection [22], but its involvement in human malaria has not been determined.

Blood MO contribute to defense, at least in part by clearance of iRBC [23],[24], but they may contribute to pathology [25],[26]. However the balance between the opposing effects is poorly understood, as available information about changes in blood MO phenotypes and functions during malaria attacks are still limited and sometimes conflicting. If the failure to control infection at a very early stage of infection is related to a lack in adequate innate immune responses [27], a detailed knowledge of the parasite-host factors involved in activating and regulating innate immune responses may illuminate how the host innate immune system responds to and/or is manipulated by *Plasmodium* infections [28],[29].

We hypothesized that discrete MO subpopulations were induced during malaria infections and associated with anti-parasitic activity. In the present study we carried out *ex vivo* studies of blood MO phenotypes and of the *in vitro* functional activities of the circulating myeloid cells found during acute episodes of uncomplicated malaria attacks in patients living on the Thai-Burmese border. We found that the balance between different blood MO subsets varied dramatically among patients, was associated with different functional anti-parasite activity by ADCI and with levels of peripheral blood parasitemia.

## Results

### Inflammatory blood MO are increased during acute uncomplicated malaria infections

**Figure 1:**
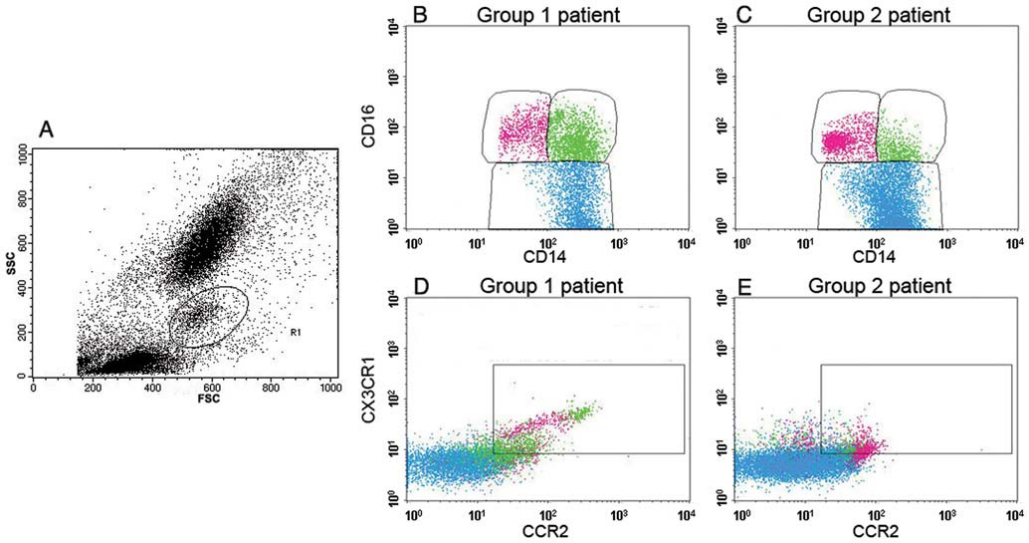
MO subsets identified in patients with acute uncomplicated malaria. (A) FSC/SSC parameters used to gate blood MO among other leukocytes. (B and C): “Classical” MO (CD14^hi^ CD16^−^ cells, blue dots), “intermediate” MO (CD14^hi^ CD16^+^ cells, green dots) and “pro-inflammatory” MO (CD14^dim^ CD16^+^ cells, pink dots) staining found in the blood of Thai patients with acute uncomplicated clinical malaria are shown. (B) In some patients, defined as Group 1, CD14^hi^ CD16^+^ intermediate MO dominated. (C) In other, clinically similar patients, and corresponding to Group 2, CD14^dim^ CD16^+^ pro-inflammatory MO dominated, CD14 FI was weak and cells were scattered. (D and E) Respective percentages of CCR2 and CX3CR1 positive cells found in each of the three blood MO subsets.

The FSC/SSC gating parameters used to distinguish between the proportions of the different leukocyte subsets and select the MO area are shown in Figure 1A. Three different MO subsets were identified in patients with acute uncomplicated malaria by CD14 and CD16 expression levels (Figure 1B and 1C). Classical MO display CD16 fluorescence intensity (FI) of less than 20 (Figure 1B and 1C). MO with a CD16 FI higher than 20 were considered as “inflammatory” MO. The CD14 expression levels defined two subsets of CD16^+^ MO: “intermediate” MO (CD14^hi^ CD16^+^) displayed a CD14 FI greater than 100, whereas “pro-inflammatory” MO (CD14^dim^ CD16^+^) had a CD14 FI of less than 100 (Figure 1B and 1C).

In healthy, malaria-exposed individuals resident on the Thai-Burmese border, the mean number of leukocytes was 6,245±1,191 µl^−1^ and that of MO was 371±133 MO µl^−1^. Only limited percentages and numbers of CD16^+^ MO were detected and most cells belonged to the classical MO subset with a mean CD14 FI of 126.1. Surprisingly, 98.4% of total blood MO of the healthy malaria-exposed individuals were TREM-1 positive (Table 1), indicating that MO from these individuals were activated.

**Table 1:** Percentages and absolute numbers of MO subsets found in patients during acute uncomplicated malaria attacks.

Donors	Categories	Mean±SD monocyte subsets (%) and mean±SD MO numbers per microliter			TREM-1+	
		Classical	Intermediate	Pro-inflammatory	% cells	FI
Malaria-exposed individuals	Healthy (n = 10)	91.4±2.5	1.6±1.2	4.6±1.9	98.4±1.7	46.2±5.8
		339±124	7±5	17±8	364±127	
Acute uncomplicated	Patients (n = 76)	48.9±19.5***	14±9.5***	34.8±18.1***	94.5±9.2	73.8±37.2***
		287±195	83±84***	205±169***	559±310*	
	Group 1 Patients (n = 19)	57.1±21.3***	12.9±9.5**	27.6±19.8***	92.3±11.1*	58.9±30.3
		360±242	80±81***	146±124***	551±290	
	Group 2 Patients (n = 57)	46.4±18***	14.4±9.5***	37.1±17.0***	95.3±8.5*	78.6±38.2**
		260±169	84±85 ***	227±179***, ^§^	562±318	

Percentages and numbers of the different MO subsets and TREM-1 positive cells found in 10 healthy malaria-exposed individuals and in the two groups of patients with clinically defined acute uncomplicated malaria attacks.

Values correspond to the mean percentages and/or numbers±1SD and to the geometric mean of Fluorescence Intensity (FI). The differences between results were tested by non-parametric 1 way median test between healthy malaria-exposed individuals (n = 10) and Group 1 (n = 19) or Group 2 patients (n = 57). Significance levels are indicated by star symbols as follows: * when *p*<0.05 and >0.01; ** when *p*<0.01 and >0.001, and *** when *p*<0.0001. Results of statistical tests between the two groups of patients are indicated by circle symbol as follows: ^§^ when *p*<0.05.

In patients with acute uncomplicated malaria attacks, the mean number of leukocytes was 8,249±3,104 µl^−1^ and that of MO was 591±324 µl^−1^. In contrast to the healthy malaria exposed individuals, the circulating MO sub-populations of patients were characterized by a 1.9 fold decrease in the percentage of classical MO subset, a 8.7 fold increase in the intermediate MO subset and a 7.6 fold increase in the pro-inflammatory MO subset (Table 1). Thus the proportion and the absolute number of both intermediate and pro-inflammatory MO subsets increased during acute malaria infection. Moreover, MO from patients showed a significantly increased expression levels of TREM-1, a marker of inflammatory MO [30], compared to the healthy malaria-exposed controls (Table 1). Taken together, these results showed a clear indication of an increase in inflammatory MO subsets during acute uncomplicated malaria.

### CD14^hi^ and CCR2^+^CX3CR1^+^ markers characterize two groups of patients with acute uncomplicated malaria

The mean level of CCR2 and CX3CR1 expression on the total blood MO was three times higher in patients with acute uncomplicated malaria (18.5±1.5%) than in healthy malaria exposed individuals (6.0±4.8%). Furthermore, it appeared that the expression of CCR2 and CX3CR1 in the CD14^hi^ MO could discriminate two groups of patients. In healthy malaria exposed individuals, the percentage of CD14^hi^ CCR2^+^CX3CR1^+^ MO was low (mean value: 5.23±4.49%, 95% CI: 1.84–8.62). Using the upper 95%CI level of these healthy individuals, we could divide the 76 malaria patients into two groups, illustrated by representative examples (Figure 1D and 1E).

In Group 1 patients (19/76, 25%), the mean percentage of CD14^hi^ CCR2^+^CX3CR1^+^ MO was 19.76±9.99% (Figure 1D), *i.e.* almost 3.78 fold higher than in healthy individuals. Compared to healthy malaria-exposed individuals, these patients displayed a 1.6 fold decrease in the mean percentage of classical MO, but a 8.1 fold increase in the mean percentage of intermediate MO and a 6 fold increase in the mean percentage of pro-inflammatory MO (Table 1).

In Group 2 patients (57/76, 75%), the mean percentage of CD14^hi^ CCR2^+^CX3CR1^+^ MO was 3.51±2.42% (Figure 1E), *i.e.* similar to that found in healthy controls. Compared to healthy malaria-exposed individuals, patients in this group showed a 1.97 fold decrease in the percentage of classical MO, an 9 fold increase in the percentage of intermediate MO and an 8 fold increase in the percentages of pro-inflammatory MO. Group 2 patients had 2.6 times more pro-inflammatory MO than intermediate MO , *versus* 2.1 in group 1 (Table 1). The highest mean percentage and mean absolute number of CCR2^+^CX3CR1^+^ total blood MO were present in Group 1 patients (Table 2); in addition, the mean percentage and absolute number of CCR2^+^CX3CR1^+^ MO were significantly greater in Group 1 patients compared with Group 2 patients among the classical and intermediate Mo but not in the pro-inflammatory subset (shown in Supporting Information as SI Table 1), all these findings being consistent with the definition of the two groups.

**Table 2:** Phenotypic characterization of blood MO from healthy malaria-exposed individuals and patients with acute uncomplicated malaria.

Surface markers	Mean±SD total blood MO % and Mean±SD MO numbers per microliter		
	Healthy malaria-exposed donors	Acute malaria patients	
		Group 1	Group 2
CD56	60±27.8	37.3±34.4	44±32.4
	222±147	221±266	264±287
mIFN-γ	54.4±29.2	30.5±31.5	31.1±27.7
	202±147	173±252	204±261
mTNF-α	13.4±17.5	13.7±11.7	16.9±18.9
	46±42	71±58	107±157
CCR2+ CX3CR1+	6.0±4.8	30.8±14.1***	14.5±10.4 ***, ^§^
	24±17	186±130***	85±87**, ^§^

The phenotypes of blood MO from healthy malaria-exposed individuals and from the 2 groups of patients with acute uncomplicated clinical malaria attacks were determined by flow cytometry. Values correspond to the mean percentages ±1SD and the differences between results were tested by non-parametric 1 way median test between healthy malaria exposed individuals (n = 10) and Group 1 (n = 19) or Group 2 patients (n = 57). Significance levels are indicated by star symbols as follows: ** when *p*<0.01 and >0.001, and *** when *p*<0.0001.

Results of statistical tests between the two groups of patients are indicated by ^§^ when *p*< = 0.01

Finally, we noted that MO of Group 1 patients were comparable to those of healthy individuals with regard to cell size and granularity. In contrast, MO of Group 2 patients had a greater size and a higher granularity than MO of Group 1 patients while expressing lower levels of CD14 (Figure 1C).

### Expression of membrane bound IFN-γ and transmembrane TNF-α

We examined the expression of CD56, membrane-bound IFN-γ and transmembrane TNF-α (mIFN-γ and mTNF-α) in the MO and their major subsets in the peripheral blood of patients with malaria and healthy controls from malaria-endemic areas. The proportions of CD56 positive and mIFN-γ positive MO were low in malaria patients compared to healthy malaria-exposed individuals, while the proportion of mTNF-α MO were similar (Table 2). Within the 3 main subsets of ‘intermediate’, ‘pro-inflammatory’ or classical MO, there were major differences between Group 1 and 2 patients concerning the markers CCR2^+^CX3CR1^+^, as expected (Supporting Information: see Table S1). In the classical MO, low percentages of CD56, mIFN-γ, and mTNF-α positive MO were observed in both groups of malaria patients, particularly in Group 2 patients, compared with the control group. In contrast, among the intermediate and pro-inflammatory MO there was a very high percentage of CD56 and mIFN-γ positive MO, and to a lesser extent, a high percentage of mTNF-α positive MO particularly in Group 2 patients, compared with the control group. However, there were no significant differences detectable between the two groups of patients regarding the CD56, mIFN-γ and mTNF-α labeling of the three MO subsets, as summarized in Table S1.

### Plasma cytokine levels are increased in the 2 groups of patients

As no difference in the surface staining of MO was found between the two groups of patients, we tested if they differed with regard to circulating levels of cytokines or chemokines, but this was not the case.

Indeed, cytokine and/or chemokine plasma levels in patients differed from levels determined in healthy, malaria-naive individuals (Table 3). For example, in Group 1 patients, significant increases in IL-1β, IL-6, IL-10, TNF-α and MCP-1 mean concentrations were found (Table 3). In Group 2 patients, this trend was even more pronounced with mean increased levels of IL-1β, IL-6, IL-10, IL-12p70, TNF-α and MCP-1 as compared to controls.

**Table 3:** Comparison of plasma cytokine or chemokine levels found in the two groups of patients.

Types	Geometric means±SD of cytokines or chemokines (pg/ml)		
	Control individuals	Group 1 patients	Group 2 patients
IL-1β	1.6±1.3	4.36±3.23 *	3.65±2.03 **
IL-6	2.8±3.0	23.33±5.14 *	30.41±6.82 **
IL-8	34±5.1	14.69±2.04	20.23±3.81
IL-10	1.8±1.2	87.50±5.26 ***	93.76±4.25 ***
IL-12p70	1.87±1.14	1.92±1.27	2.41±1.38 ***
IFN-γ	2.19±1.18	1.90±6.48	4.41±3.18
TNF-α	1.63±1.13	2.83±2.43 **	2.47±1.59 ***
MCP-1	50.12±1.69	239.9±3.43 **	426.60±3.93 ***

The cytokine and chemokine concentrations in the serum of 13 Group 1 and 54 Group 2 patients were determined by flow cytometry (using CBA flex set®). All the available plasma samples were tested at the same time and the differences between concentrations were determined by non parametric 1 way median Tests between Group 1 or Group 2 patients. 10 control individuals (healthy malaria naïve individuals) were also tested and, as expected, cytokine or chemokine concentrations differed from those found in patients, Significance levels are indicated by star symbols as follows: * *p*<0.05, ** *p*<0.01 and *** *p*<0.001.

The concentration of IL-12p70 was marginally higher in Group 2 than in Group 1 patients but these differences fell just short of statistical significance (*p* = 0.0637).

In the 76 patients, IL-1β and TNF-α levels were strongly correlated (R^2^ = 0.957) and weaker but significant correlations were observed between IL-6 and IL-8 and between IFN-γ and IL-8 (R^2^ = 0.494 and 0.375 respectively).

### The two groups of patients defined by MO subsets differ by parasite load and the stage of circulating parasites

**Figure 2:**
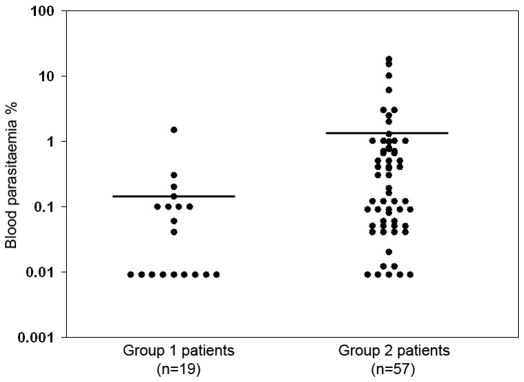
Distribution of peripheral parasitemia in patients with acute uncomplicated malaria. Individual parasitemia levels determined in 19 Group 1 and 57 Group 2 patients with clinically defined acute uncomplicated malaria attacks are shown.

We wondered if the two different distributions of MO phenotypes in patients with malaria were related to different densities of circulating parasites. We found that peripheral blood parasitemia levels at admission were higher in Group 2 than in Group 1 patients. The mean±SD parasitemia was 0.14±0.34% and 1.30±3.34% in Group 1 and in Group 2 patients, respectively (*p* = 0.0053). However, the ranges of parasitemias were wide, especially in Group 2 patients (Figure 2). So, at the individual level, the MO phenotype was not predictive of parasite densities.

In addition, the relative level of maturation of circulating parasites differed between patients. Blood parasites were exclusively at the ring stage in 63.15% of the slides (12 out of the 19) from Group 1 patients but in only 22.08% (13 out of 57 slides) of the blood smears from Group 2 patients (Pearson Chi square = 4.88, *p* = 0.03). These observations led us to compare the anti-parasite functional capacities of peripheral blood MO in patients from Group 1 and 2.

### Blood MO from Group 1 and Group 2 patients differed in antibody-dependent parasite growth inhibition assays

**Figure 3:**
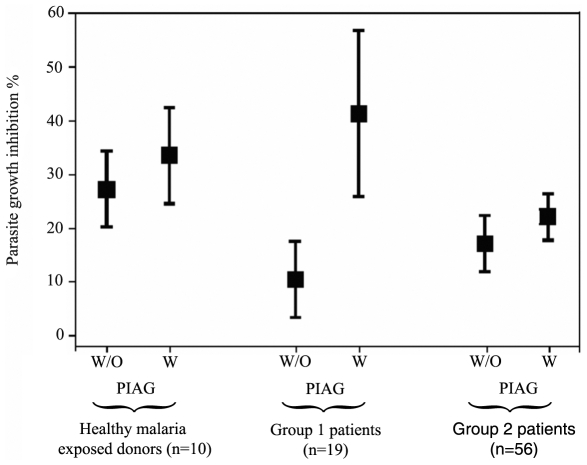
Growth inhibition of parasite cultures by MO from patients with malaria. The percentage of *P. falciparum* growth inhibition is shown firstly, with MO but without (w/o) purified immune IgG and secondly, in the presence of MO plus purified immune IgG (PIAG). Data represent the mean percentages of parasite growth inhibition±95% CI.

MO from healthy malaria-exposed individuals and from malaria patients were compared for their *in vitro* inhibitory effect on the growth of *falciparum* parasites with and without IgG (Figure 3). When MO of 12 healthy European malaria-naïve individuals were used alone without IgG, they exerted no or only very limited direct growth inhibition effects (mean±SD: 3.8±4.3%). In contrast, MO from 10 healthy malaria exposed individuals inhibited parasite growth by 27.5±11.0%, hence, they appeared to be 7.2 fold more active than MO from healthy but malaria-naïve individuals. It was notable that, the direct inhibitory effect of MO from patients was lower than that of the healthy malaria-exposed individuals, being 10.8±11.7% and 17.4±16.0% for patients from Group 1 and Group 2, respectively. However, in the presence of immune IgG (PIAG), to measure ADCI, the mean parasite growth inhibition was 40.77±4.22% (95%CI: 32.35–49.19%) with MO from Group 1 patients and 22.38±2.48% (95% CI: 17.43–27.33%) with MO from Group 2 patients (*p* = 0.0174).

The specific growth inhibitory index (SGI) was calculated for all these ADCI assays. In healthy malaria-exposed individuals, the mean SGI was 7.99±7.93% [95% CI; 2.31–13.67%]. For Group 1 patients the mean SGI was 29.54±30.74% [95% CI; 14.72–44.35%] and it was significantly greater than the SGI from MO of Group 2 patients (3.41±5.78% [1.85–4.97%] (*p*<0.0001)). In conclusion, an antibody and monocyte-dependent inhibition of parasite multiplication (ADCI) was observed with the MO from Group 1 patients only.

In order to limit a residual effect of parasite-specific and membrane bound immunoglobulins, MO were thoroughly washed before performing the inhibition experiments. Furthermore, when anti-parasite antibodies titers were determined, no significant differences were found between Group 1 and Group 2 patients as defined by their MO subsets (see above). Anti-*P. falciparum* Arbitrary Units AU for total IgGs were 1.74±1.14 and 1.81±1.04 (*p* = 0.851), for IgG_1_ 3.9±4.08 and 5.28±3.52 (*p* = 0.5551) and for IgG_3_ 5.42±5.76 and 4.92±45.03 (*p* = 0.552) in Group 1 and Group 2 patients, respectively. Thus differences in anti-malarial antibody titers were very unlikely to have been responsible for the observed differences in parasite growth inhibition.

### In patients, ADCI activity was high when the percentage of CD14^hi^ CCR2^+^CX3CR1^+^ MO was elevated

As mentioned above, an ADCI effect was observed with MO from Group 1 patients only. More precisely, 10 out of 19 Group 1 patients had ADCI results above 20%, a situation associated with significantly low parasitemias (Figure 2). Therefore, Group 1 patients from this malaria endemic area of Thailand, initially delineated exclusively on the basis of a singular MO phenotype, also had a distinct difference in functional ADCI anti-parasite biological activity.

To further characterize the phenotype of MO with ADCI activity, MO from Group 1 patients were analyzed in greater detail, by comparing the 10 individuals with MO active in ADCI with the 9 individuals with no ADCI activity. As shown in Table 4, MO activity evaluated by ADCI was simultaneously associated with a high percentage of classical MO subset and with a low percentage and a low absolute number of pro-inflammatory MO subset. MO from patients with high levels of ADCI contained as much as 72% of classical MO, and these MO with strong ADCI activity showed significantly greater expression of CCR2, CX3CR1, mTNF-α, CD56, and mIFN-γ than MO with no ADCI activity (Table 4). These results suggested that, in patients, ADCI activity is associated with a particular MO subset, expressing CCR2 and CX3CR1, mTNF-α, CD56 and mIFN-γ.

**Table 4:** Phenotypic characterization of blood MO from group 1 patients with or without ADCI activity.

Categories	Surface markers%	10 patients with ADCI activity	9 patients with no ADCI	*p* values
* SGI%		51.6±27.1	5.1±4.2	*p* = 0.0001
Parasitemia%		0.018±0.029	0.28±4.47	*p* = 0.0008
Classical MO	CD14^hi^ CD16^−^	72.1±14.9%	38.3±13.3%	*p = *0.0035
		484±255 /µl	223±137 /µl	*p* = 0.0427
	CD56	22.7±25.5%	0.5±0.9%	*p* = 0.0427
		131±217 /µl	4±8 /µl	*p* = 0.0427
	mIFN-γ	21.6±27.8%	0.2±0.4%	*p* = 0.0220
		121±223 /µl	2±4 /µl	*p* = 0.0220
	mTNF-α	2.1±2.8%	0	*p* = 0.0001
		15±22 /µl	0	*p* = 0.0016
	CCR2^+^ CX3CR1^+^	16.9±6.8%	5.4±3.1%	*p* = 0.0427
		102±59 /µl	29±24 µl	*p* = 0.0085
Intermediate MO	CD14^hi^ CD16^+^	11.8±9.5%	14.0±10.0%	*p* = 0.509
		74±72 /µl	87±94 /µl	*p* = 0.814
Pro-inflammatory MO	CD14^dim^ CD16^+^	13.5±7.9 (%)	43.9±1 7.1 (%)	*p* = 0.0008
		75±45 (/µl)	216±131 (/µl)	*p* = 0.0095

*: SGI% = The parasite specific growth inhibition (i.e. the ADCI effect) was calculated as indicated in the Material and Methods section.

The 19 patients from Group 1 were tested with regard to ADCI activity.

Ten patients had a positive ADCI activity (with SGI>20%), and 9 patients had a negative ADCI activity. MO phenotypes were tested and compared for various surface marker expressions. Differences in staining were found in the classical and the pro-inflammatory MO subsets as indicated. The statistical analyses were performed by non-parametric 1 way median tests.

## Discussion

We have shown for the first time distinct differences in the MO subsets in patients with acute malaria and healthy malaria-exposed controls. Furthermore, we have shown that patients with high numbers of CD14^hi^ CCR2^+^CX3CR1^+^ MO had lower parasitemias than patients with low numbers of CD14^hi^ CCR2^+^CX3CR1^+^ MO. Our results suggest this is a causal association as circulating MO expressing CCR2, CX3CR1, CD56, mTNF-α and mIFN-γ are not only associated with lower parasitemias but also are associated with strong ADCI activity.

Since the historical observations of Talliaferro, MO and macrophages have been largely looked upon as phagocytic cells in malaria infections, clearing parasites with or without opsonizing antibodies. After a long era which concentrated almost solely on the role of lymphocytes in host immune responses, conceding an additional role to MO as antigen-presenting cells, this myeloid lineage are now perceived as comprising a very wide number of distinct cell subsets, with distinct phenotypes and numerous important functions. Nevertheless, the precise functional correlates of MO subsets defined by surface phenotype are far less defined than the functions of lymphocyte subsets.

Given the outstandingly high foreign parasite mass circulating in the blood during acute *falciparum* malaria and the strong induction of IFN-γ by both pre-erythrocytic stage and by blood-stage parasites, potentially leading to a very strong pro-inflammatory stimulus, there are ample reasons to analyze changes in MO numbers, phenotypes and function during malaria. We have shown substantial changes in MO phenotypes in non-infected populations living in endemic areas and also changes in MO phenotypes in acute uncomplicated malaria cases in adults. Our results reveal several biologically significant modifications in phenotype, cytokine-chemokine expression and activity of patients' MO. In particular, we have shown that infection in patients with a similar clinical presentation can result in the induction of two distinct MO phenotypes that can be distinguished by a single set of markers, but show quite different, possibly opposing, biological anti-parasite activity. Blood MO of malaria-exposed but non-infected individuals were significantly more mature and more activated than those from Europeans controls. In our study MO from malaria-exposed individuals showed markedly increased expression of CD56, mIFN-γ and mTNF-α, TREM-1 and HLA-DR, in the classical MO subset. The CD56 and CD33 positive MO have been associated with higher HLA-DR expression [31]. Others have described an increase in the CCR5 expression of circulating MO in Italian expatriates living in malaria endemic areas [32]. These studies do not determine the origin of these changes in MO sub-populations. It may be that these changes reflect the long term consequences of previous malaria episodes, or are, as proposed by others, due to so-called “environmental factors”, such as exposure to mosquito bites and saliva [32] or even induced by increased IFN-γ stimulated by chronic viral infections prevalent in malaria-endemic areas [33]. Whatever the reason for the induction of these cells, the distinctive MO phenotype associated with infection led us to analyze the functional associations of MO subsets during *falciparum* malaria.

As expected blood MO from acute malaria patients had several characteristics of inflammatory MO, with an overall increase in the percentage of CD16^+^ MO, and in secreted inflammatory cytokines, with a substantial and significant decrease in the percentage of classical MO and a corresponding increase in both intermediate and pro-inflammatory MO. Patients' MO had significantly lower percentages of CD56 and mIFN-γ and higher percentages of CCR2^+^CX3CR1^+^ than malaria-exposed individuals, and high expression of the inflammatory marker TREM-1.

Given the large range of markers we used to define MO subsets, results are difficult to relate to other studies. However, our data are consistent with more limited previous studies. In Malawi, increased CD16^+^ MO populations were described during malaria infection [34]. In our study, among 76 patients with acute uncomplicated malaria studied the major finding was the characterization of two groups of patients with different MO phenotypes. Group 1 patients, characterized by an elevated percentage of CD14^hi^ CCR2^+^CX3CR1^+^ MO, had a low mean parasitemia. Group 2 patients, characterized by a percentage of CD14^hi^ CCR2^+^CX3CR1^+^ MO similar to that of healthy malaria exposed individuals, had a pro-inflammatory phenotype, a trend for higher levels of plasma cytokines, and a 10 fold higher mean peripheral parasitemia. Finally, Group 1 and 2 MO differed in their parasite inhibition activity in cooperation with effective parasite-specific antibodies.

These observations raise two questions. First, is there a causal relationship between those parameters and, second, are these characteristics genetically determined, or do they reflect different states in the course of a *falciparum* clinical attack? It is indeed tempting to speculate that patients developing the predominantly anti-inflammatory MO phenotype have lower parasitemia because their MO are highly effective at achieving parasite inhibition in cooperation with antibodies as in the ADCI mechanism, while patients with the pro-inflammatory phenotype have higher mean parasite densities as their MO are completely ineffective at inhibiting parasite growth. The lack of a complete correspondence between ADCI activity and parasitemia loads could be due, at least in part, to the well-known poor association between peripheral blood parasitemia and total parasite burden [35]. But it may also be relevant that the stage of circulating parasite differed between the two groups of patients. The presence of mature parasites in the blood (which was more frequent in Group 2 patients) was consistent with similar indications independently obtained recently in the same malaria endemic area [36].

There may be genetic factors that determine the degree and direction of MO development during acute infection. Genetic control of several innate and adaptive immune responses is documented and individuals differ markedly in their susceptibility to systemic infection, inflammatory states and in their ability to mount a rapid pro-inflammatory cytokine response to malaria infection [37]. However, it is also possible that the two groups may not reflect genetic differences but temporal differences in the innate response to *falciparum* parasites.

The initial rise in parasitemia can be expected to induce a very strong pro-inflammatory response that may control parasite growth. Indeed, activated macrophages are essential for host survival in animal models of malaria [38]. However inflammatory cytokines and free NO and O_2_ radicals triggered by parasite material could also contribute to much of the pathology seen in malaria [39], so that it is vital that anti-inflammatory responses are induced to avoid excessive tissue damage [40]. Therefore, it is possible that the initial pro-inflammatory response may be represented in Group 2 patients and the subsequent anti-inflammatory regulatory response represented by Group 1 patients. Whether the intriguing observation of lower parasitemia seen in Group 1 is the result of a previous pro-inflammatory state or more directly of functionally more effective ADCI activity in MO, cannot be determined. Additional studies particularly with follow-up of patients in different clinical presentations and epidemiological situations are now obviously required.

Results from *falciparum* growth inhibition studies are interesting and pose many questions. Antibodies effective in the MO-mediated antibody-dependent ADCI mechanism have been extensively studied [41]–[43] but these studies have always used MO from healthy European blood donors. Whether MO isolated from patients during a malaria attack also retain ADCI activity had not been addressed. Here, we have shown that some but not all patients' MO can exert strong ADCI activity. The low or absent ADCI activity of pro-inflammatory MO from Group 2 patients is consistent with our previous observations that activated MO from donors suffering from a viral infection or activated macrophages derived from normal blood MO by adherence *in vitro* over 48–72 hours, had low activity in ADCI assays [44]. Conversely, MO which are activated by classical pro-inflammatory stimuli may exert a direct, antibody-independent, anti-parasite effect seen in MO from exposed endemic area donors and here to a lesser extent in MO from Group 2 patients, in contrast to the low direct, antibody-independent anti-parasite effect of MO from healthy European donors. Though non-opsonic clearance of iRBCs do occur [45],[46], the very low MO/RBC ratio in ADCI assays suggests that mediators released by activated MO, including chemokines, cytokines and free radicals, must play a more prominent role.

Above all, the characterization of patients MO with distinct differentiation and activation states led us to identify far more precisely the features of the blood MO subset most effective at mediating ADCI. Our observation of the MO subset with increased CCR2^+^CX3CR1^+^, mTNF-α and CD56 expression as having critical ADCI activity, complements our *in vitro* studies showing that the co-activation of both CD32 and CD16 was required for ADCI [47]. No similar increase in double positive MO has been reported in other infectious diseases.

In conclusion, MO and macrophages have multiple functions during patent *falciparum* parasitemia. In response to parasite-driven signals, they adapt to directly reduce parasite loads in an antibody-independent manner, to initiate and later to regulate adaptive immune responses, to remove infected RBC by antibody-dependent opsonization and by the ADCI mechanism. The present study provides several new valuable insights into both the initial and last steps of this series of actions. In patients with acute malaria, we have shown two distinct MO phenotypes with correspondingly different ADCI phenotypes. This work complements decades of studies concentrating on T-cells and antibodies, and paves the way for further characterization of dynamic studies of MO subsets and functions during malaria infection.

## Materials and Methods

### Study site and individuals tested

We studied indigenous malaria patients living in Thai-Burmese border. Seventy six patients (27.7±10.1 years old) with uncomplicated *falciparum* malaria, i.e. with fever >37.5°C but without features of severe or complicated disease and with a mean parasitemia of 1.01±2.93% and 10 healthy malaria-exposed individuals (26.2±6.9 years old) with negative blood smears were enrolled in this study during the 2007 transmission season. These patients had not been treated before admission, had no other cause of disease than malaria identified at enrollment and they recovered after anti-malaria treatment. Five to 10 ml of blood were taken from patients or healthy individuals. Written informed consent was obtained from all participants after detailed explanations of the studies in the local language. This protocol was approved by the Research Ethics Committee, Faculty of Medicine Siriraj Hospital, Mahidol University, Thailand. Blood samples from 12 malaria naïve individuals (29.42±5.14 years old) living in France were obtained from the blood bank in Paris, with the consent of the Pasteur Institute Ethical Committee, Paris, France.

### Parasite cultures

The *P. falciparum* laboratory strain TM267 [48] was obtained from Dr. Kesinee Chotivanich (Mahidol University, Thailand) and previously used for other studies. TM267 is a knobless parasite strain which is unable to bind endothelia and specific host ligands. Parasite cultures were regularly checked by PCR to exclude mycoplasma contamination and maintained in Group O^+^ human RBC at 37°C with 5% CO_2_ suspended in RPMI medium 1640 (Sigma, Germany) containing either 10% heat-inactivated AB^+^ human serum (for routine cultures) or 10% Albumax (GIBCO, USA) (before ADCI experiments).

### Monocyte surface staining

The phenotype of cells were determined by direct immunofluorescence with monoclonal antibodies (mAbs) to CD14, CD16, CD33, HLA-DR, CD56, CD16, IFN-γ-, and TNF-α conjugated with fluorescein isothyocyanate (FITC), phycoerythrin (PE), and peridinin chlorophyll protein (PerCP) (BD Bioscience, Oxford, UK) and mAbs to CCR2, TREM-1 (Serotec, Oxford, UK), and CX3CR1 (MBL, Nagoya, Japan) conjugated with APC, PE, and FITC, respectively, following the manufacturer's protocols. Isotype matched controls were used in all experiments.

Two hundred microliters of well-mixed whole blood from each patient sample was used for each analysis. Of note, the totality of the 200 µl of blood were systematically passed through the instrument, hence, between 30,000 and 60,000 blood MO of each blood sample were counted. Three color analysis was performed with CD14, CD16, and CD33 or HLA-DR or IFN-γ, or TNF-α or TREM-1 or CD56. In the latter case, only MO with low FI of CD56 were acquired. Four colors analysis was used to determine CD14, CD16, CCR2 and CX3CR1 expression. Using a FACSCalibur (Becton Dickinson, NJ, USA), MO were first identified and gated by side and forward scattering. Final gates to exclude NK cells but including CD14, CD33 and TREM-1 positive cells were then defined [21] and used throughout the study.

### Determination of parasite-specific IgG by ELISA

Total IgG and subclasses were determined by ELISA in 96 well Maxisorb® plates (Nunc, Paris, France) [49] with anti-IgG1 (clone NL16) and anti-IgG3 (clone ZG4) mAbs (Skybio, Cambridge, UK). Lysates of Plasmagel enriched mature malaria infected RBCs were used as antigen [50]. A pool of immune sera from Africa and a pool of malaria-naïve Europeans' sera were used as controls. The specific reactivity of each serum sample was expressed as a ratio (thus Arbitrary Units) obtained by dividing the OD obtained from each test serum by the mean OD obtained from 6 negative control sera.

### Antibody and monocyte-dependent inhibition of parasite multiplication (ADCI)

The ability of each patient's MO to inhibit the *in vitro* growth of parasites was evaluated either with no IgG, with IgGs purified from a pool of sera obtained from healthy donors never exposed to malaria (NIgG), or with immune IgG purified (PIAG) from a pool of 333 adults from Ivory Coast. The ADCI assays followed the protocol previously described [6], with minor modifications, as indicated below. For MO preparation, cell suspensions obtained after Histopaque were adjusted to 4×10^6^ MO ml^−1^ in RPMI 1640 without serum. Then 100 µl of cell suspension with 10% Albumax were distributed into wells of a sterile 96-well flat bottom plate (Nunc, France) and incubated for 30′ at 37°C and in 5% CO_2_. Non-adherent cells were removed by gently washing each well thrice with 200 µl of RPMI 1640 and the plates were turned upside down at the end of each washing so that only cells adhering to the bottom of the wells remained whereas most contaminant cells were eliminated. Both CD14^+^CD16^−^ and CD14^+^CD16^+^ MO remained attached and satisfactorily reflected the initial proportion of the different blood MO identified and present in each patient's blood (as illustrated in Supporting Information Figures S1 and S2). Each ADCI test used 50 µl of either asynchronized 0.5% parasite culture (in the test wells) or uninfected red blood cells (uRBC) (in the control wells) diluted at a final 2.5% hematocrit. Ten µl of either immune or normal, purified IgG or RPMI were added into the test or control wells respectively. RPMI 1640 with 10% Albumax was added to final volume of 200 µl. The parasite cultures were maintained at 37°C for 72 hours in 5% CO_2_. All tests and controls were performed in duplicate and parasitemia was assessed by flow cytometry as described [42].

### Cytokine detection

Cytometric bead arrays (CBA) (BD Biosciences, Oxford, UK) were used to measure MCP-1, IL-1β, IL-6, IL-8, IL-10, IL-12β, IFN-γ and TNF-α following the manufacturer's instructions. Plasma samples were separated from whole blood by centrifugation, kept at −20°C and tested at the same time. Standard curves for each cytokine were generated by using the reference cytokine concentrations supplied with the kits. Raw data were analyzed with the CBA Software to obtain concentration values as described by Jimenez *et al* [51].

### Statistical analysis

Univariate and multivariate analyses were carried out using Statview5®, SPSS16® or JMP® softwares. Non-parametric (one way median test) or parametric tests (t test) were used to compare the different Groups where appropriate and a *p*<0.05 was considered as significant.

## Supporting Information

Figure S1 Adherence of CD14^hi^ and CD14^dim^ MO. Figure 1A shows CD14 positive cells and Figure 1B is an overlay of light transmission microscopy and fluorescent cells (objective 60×). The white arrow shows a CD14^hi^ MO and the black arrow shows a CD14^dim^ MO.
(2.34 MB PDF)

Figure S2 Adherence of CD14^+^ (CD14^hi^ and CD14^dim^) CD16^+^ MO. Figure 2A is a light transmission field. In Figure 2B, bright cells were CD14^+^ cells and in Figure 2C, bright cells correspond to CD16^+^ cells. Figure 2D is an overlay of CD14^+^ and CD16^+^ cells (objective 40×). By this technique we could identify CD14^hi^ and CD14^dim^ MO and intermediate as well as pro-inflammatory MO (data not shown).
(3.12 MB PDF)

Table S1 Percentages and numbers of CD56, mIFN-γ, mTNF-α and double positive CCR2 and CX3CR1 expressed in each subset of blood MO. The percentages of blood MO positive for different markers were determined in each MO subset. The mean percentages ±1SD of positive blood MO were obtained after three color analysis (using anti-CD14, anti-CD16, and either anti-CD56, or anti-mIFN-γ anti-mIFN-γ or anti-mTNF-α) or after four color analysis (using simultaneously anti-CD14, anti-CD16, anti-CCR2 and anti-CX3CR1 monoclonal antibodies). The differences between results were tested by non-parametric 1 way median test between healthy malaria exposed individuals (n = 10) and Group 1 (n = 19) or Group 2 patients (n = 57). Significance levels are indicated by star symbols as follows: * when *p*<0.05 ** when *p*<0.01 and *** when *p*<0.001. Results of statistical tests between the two groups of patients are indicated by symbols as follows: § when *p*<0.05; §§ when *p*<0.001 and §§§ when *p*<0.0001.
(0.04 MB PDF)
