# Supplementary figures and images for: A Distinct Peripheral Blood Monocyte Phenotype Is Associated with Parasite Inhibitory Activity in Acute Uncomplicated Plasmodium falciparum Malaria
Source: PLoS Pathog. 2009 Oct 23;5(10):e1000631. doi: 10.1371/journal.ppat.1000631 (PMC2759288; doi:10.1371/journal.ppat.1000631)

A

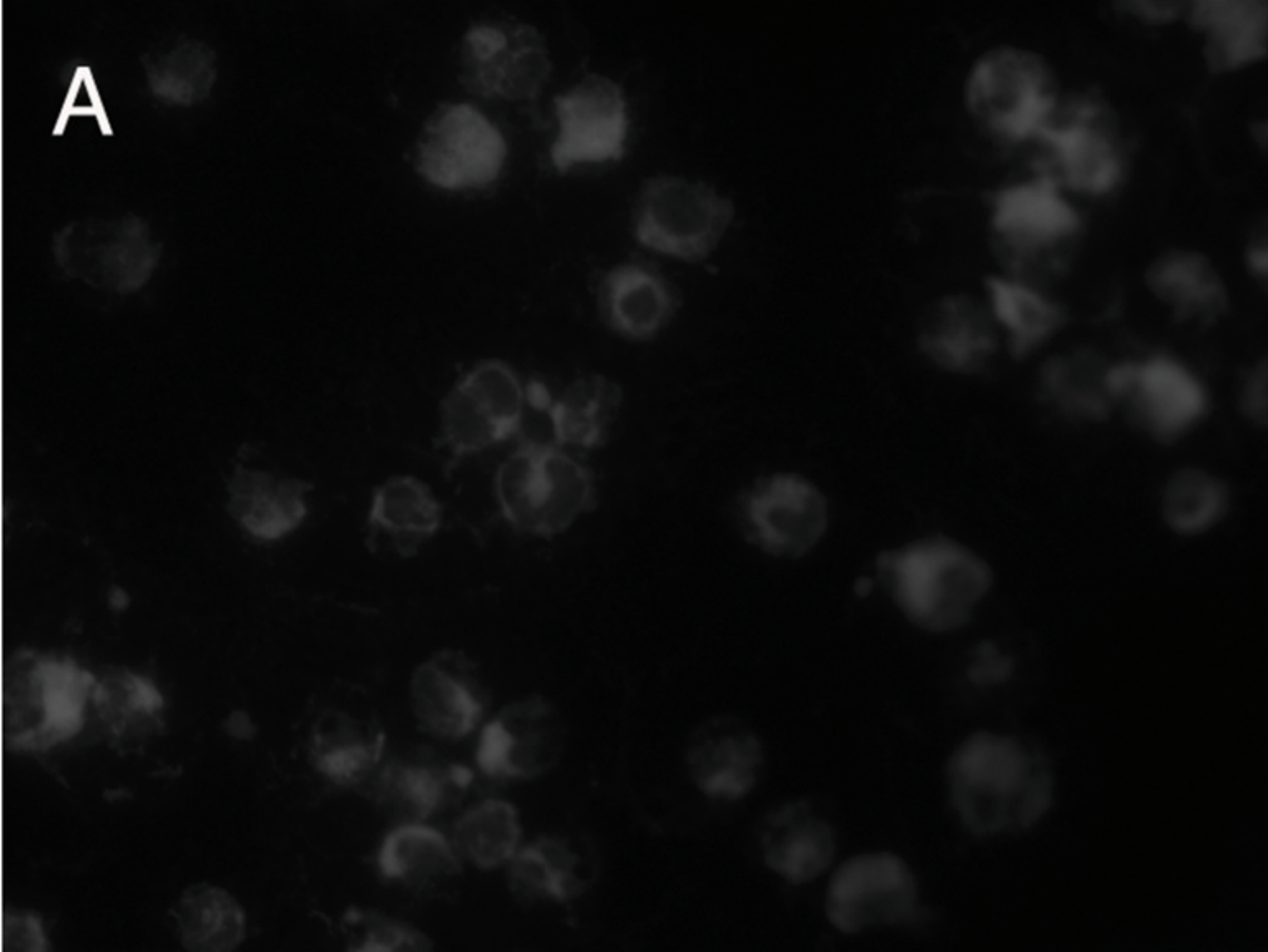

B

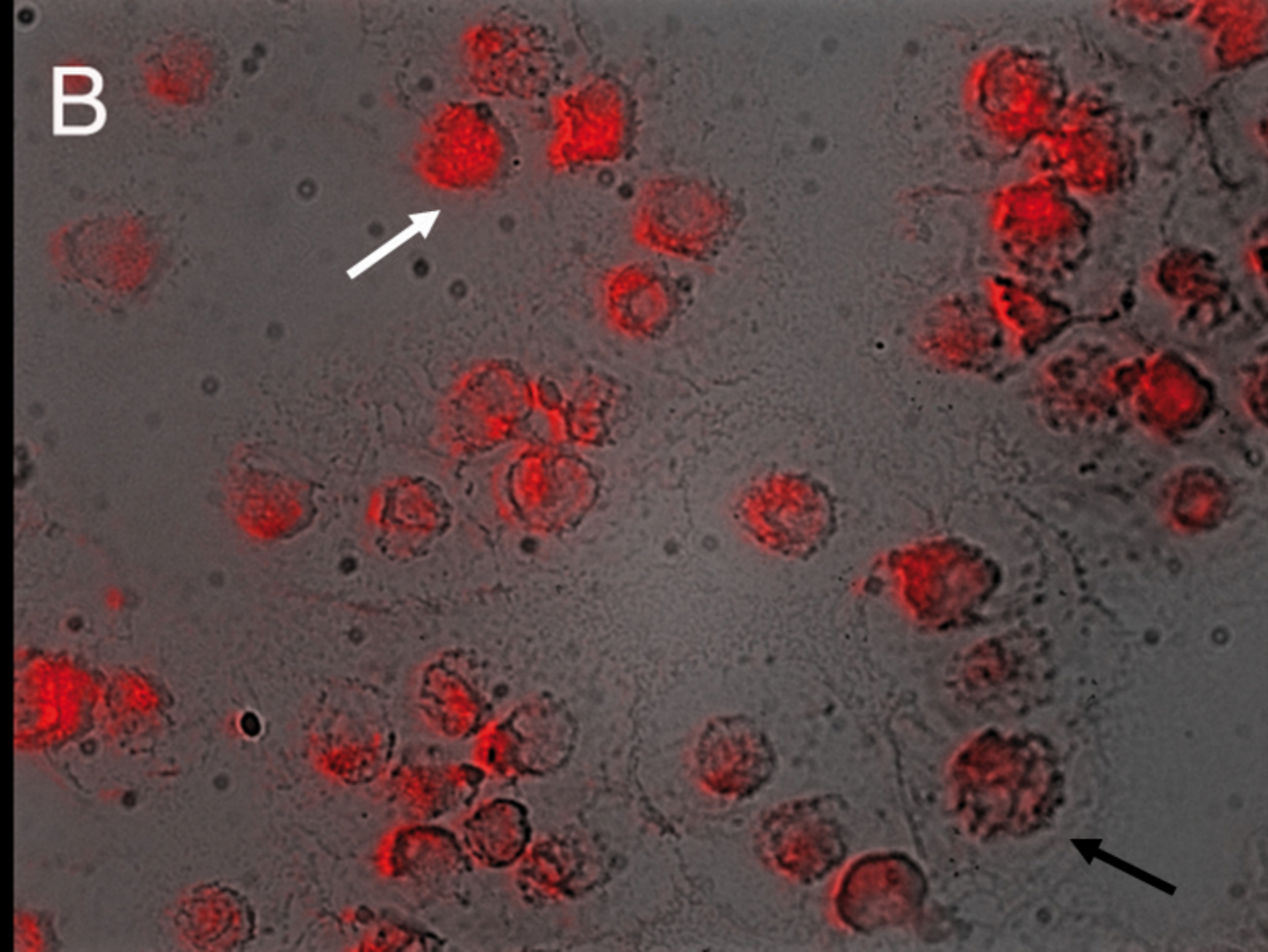

Supplement: Figure S1 — Adherence of CD14hi and CD14dim MO. Figure 1A shows CD14 positive cells and Figure 1B is an overlay of light transmission microscopy and fluorescent cells (objective 60×). The white arrow shows a CD14hi MO and the black arrow shows a CD14dim MO. (2.34 MB PDF) [file ppat.1000631.s001.pdf]

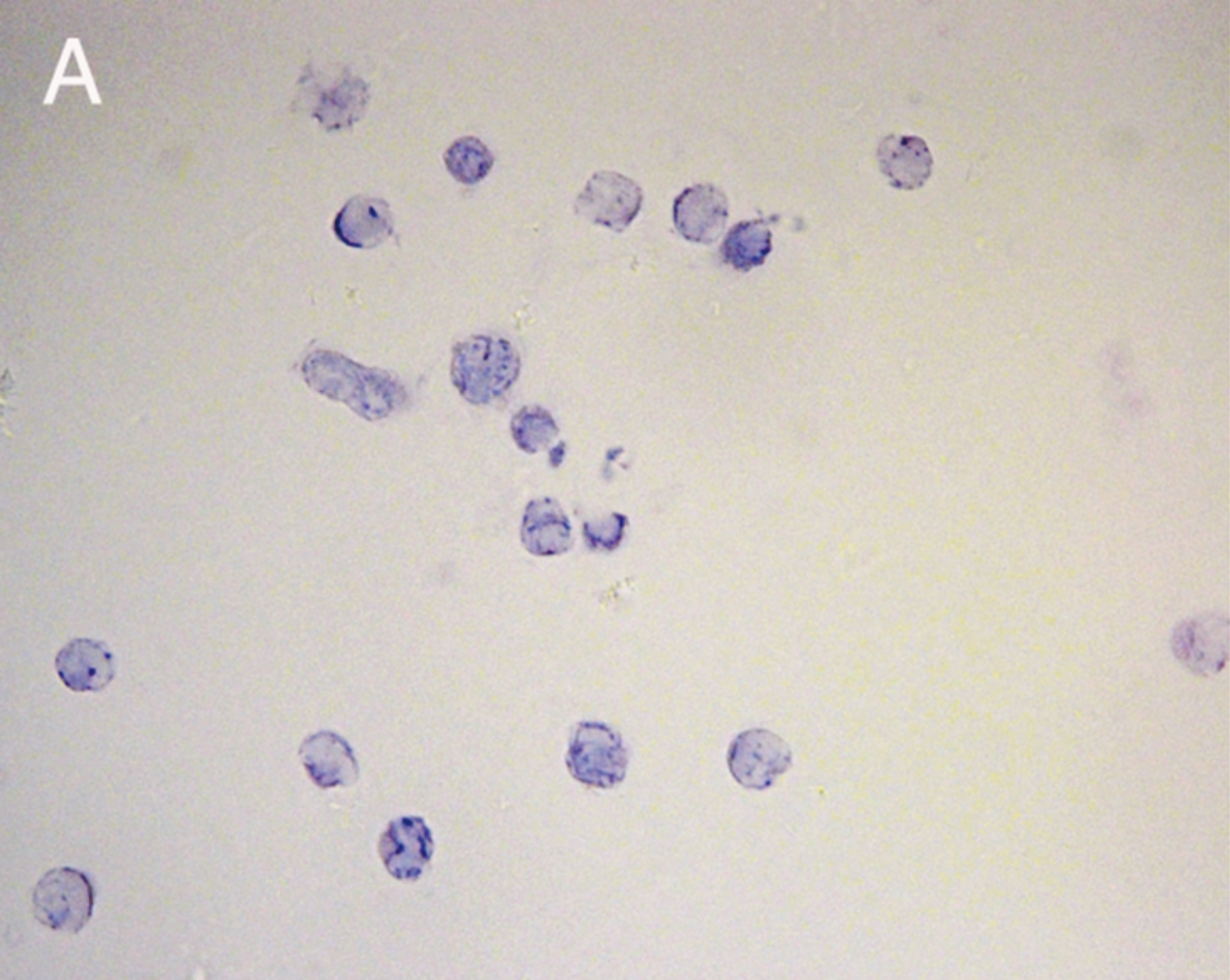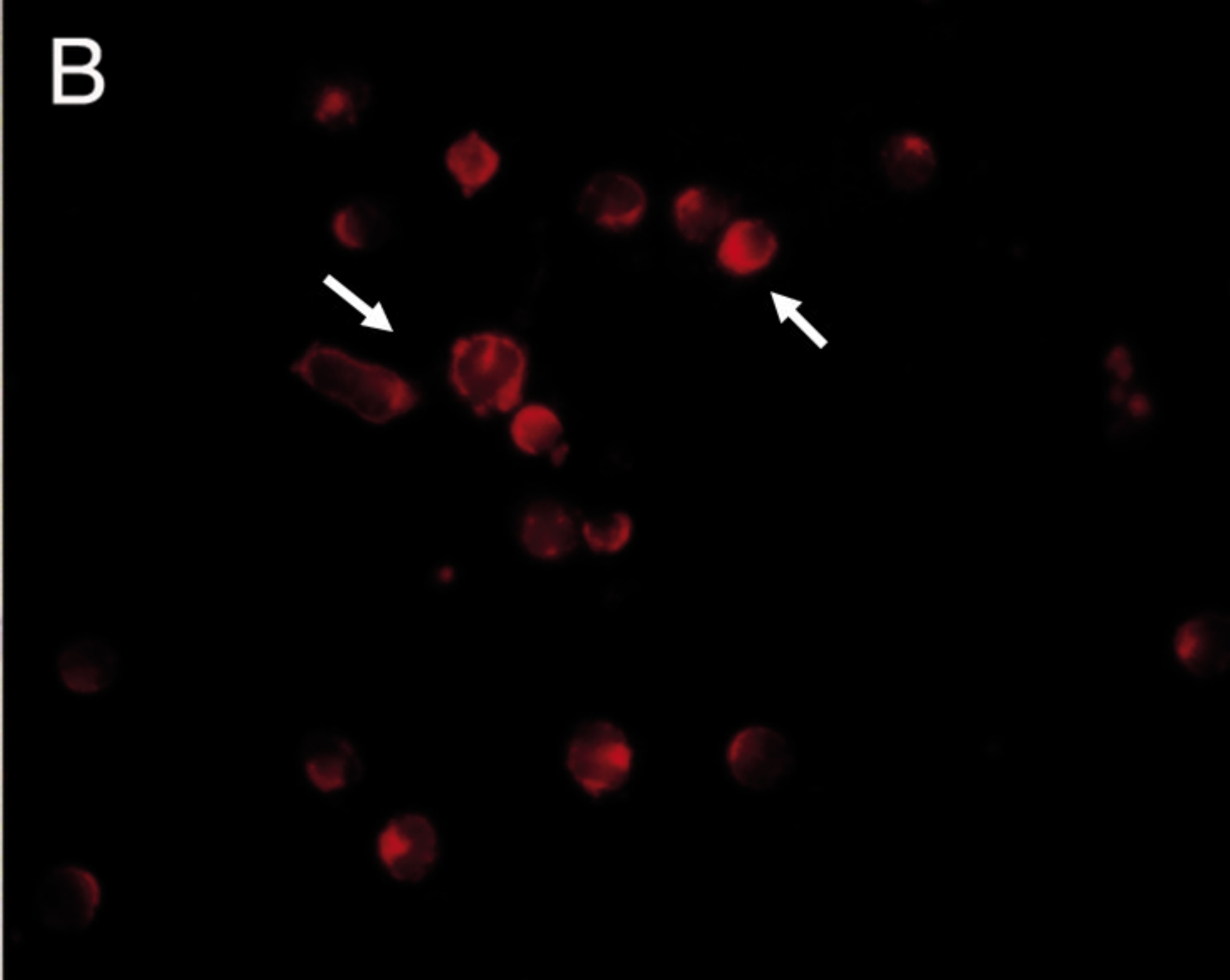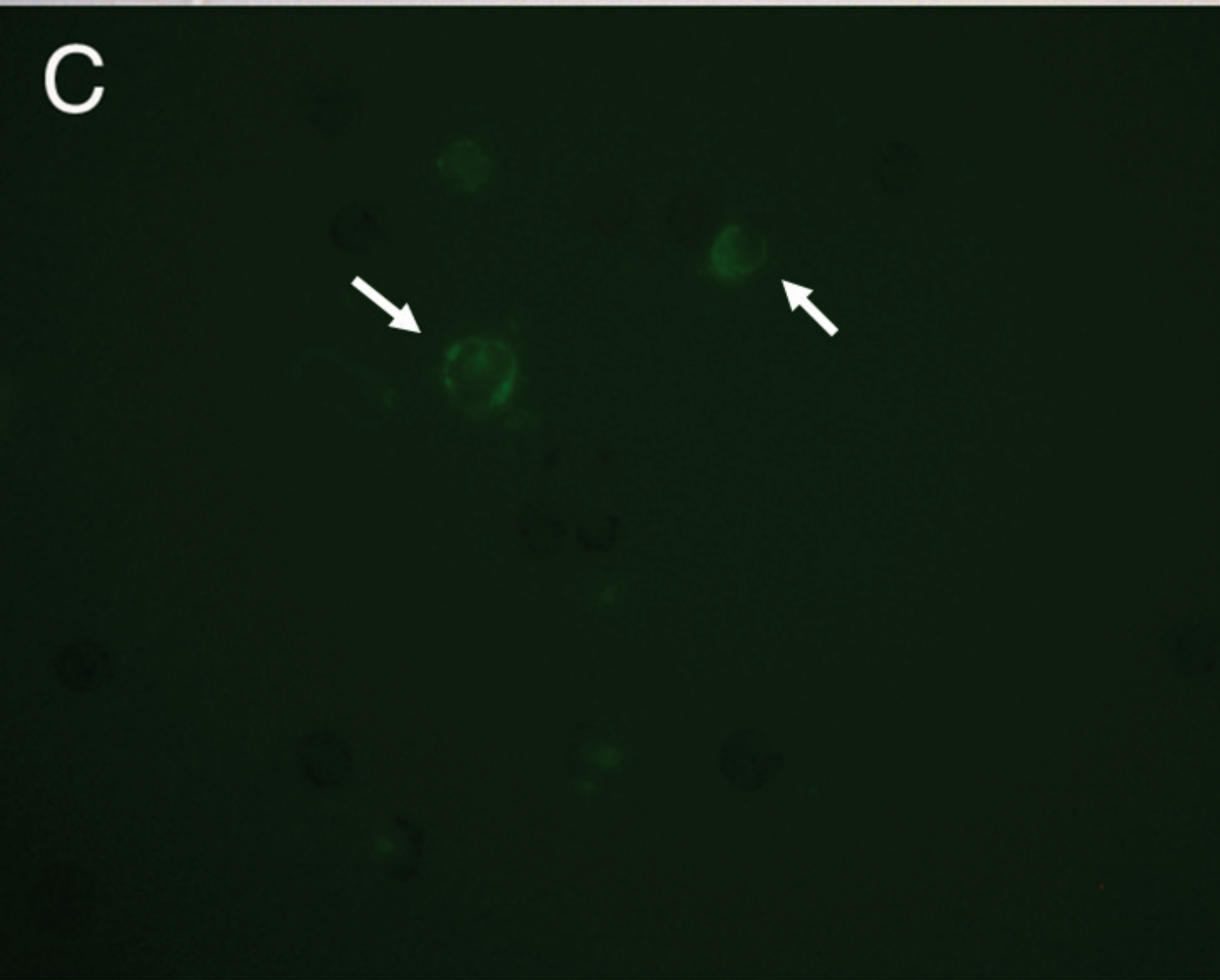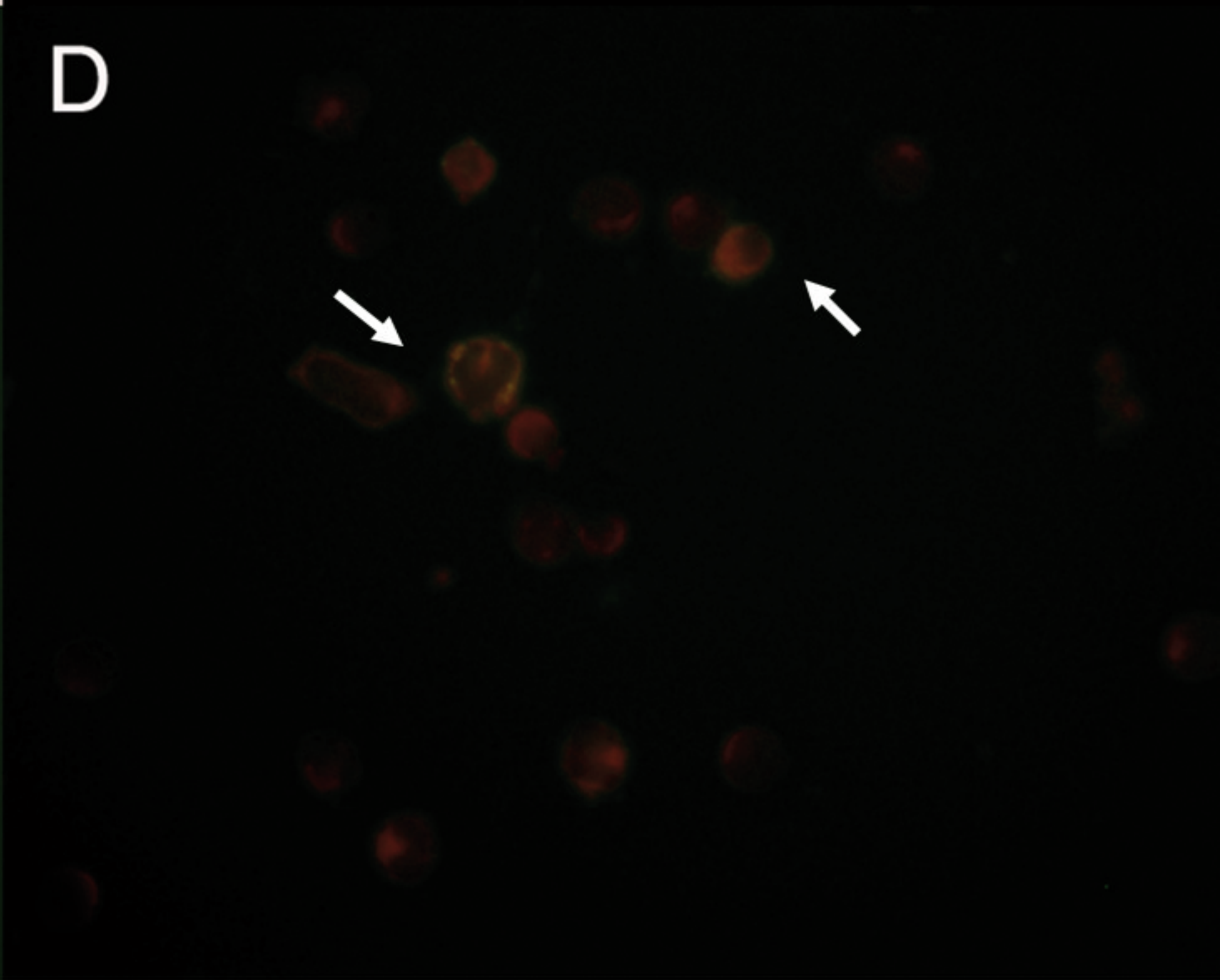

Supplement: Figure S2 — Adherence of CD14+ (CD14hi and CD14dim) CD16+ MO. Figure 2A is a light transmission field. In Figure 2B, bright cells were CD14+ cells and in Figure 2C, bright cells correspond to CD16+ cells. Figure 2D is an overlay of CD14+ and CD16+ cells (objective 40×). By this technique we could identify CD14hi and CD14dim MO and intermediate as well as pro-inflammatory MO (data not shown). (3.12 MB PDF) [file ppat.1000631.s002.pdf]
