# Supplementary material for: A Distinct Peripheral Blood Monocyte Phenotype Is Associated with Parasite Inhibitory Activity in Acute Uncomplicated Plasmodium falciparum Malaria
Source: PLoS Pathog. 2009 Oct 23;5(10):e1000631. doi: 10.1371/journal.ppat.1000631 (PMC2759288; doi:10.1371/journal.ppat.1000631)
Supplement: Table S1 — Percentages and numbers of CD56, mIFN-γ, mTNF-α and double positive CCR2 and CX3CR1 expressed in each subset of blood MO. The percentages of blood MO positive for different markers were determined in each MO subset. The mean percentages ±1SD of positive blood MO were obtained after three color analysis (using anti-CD14, anti-CD16, and either anti-CD56, or anti-mIFN-γ anti-mIFN-γ or anti-mTNF-α) or after four color analysis (using simultaneously anti-CD14, anti-CD16, anti-CCR2 and anti-CX3CR1 monoclonal antibodies). The differences between results were tested by non-parametric 1 way median test between healthy malaria exposed individuals (n = 10) and Group 1 (n = 19) or Group 2 patients (n = 57). Significance levels are indicated by star symbols as follows: * when p<0.05 ** when p<0.01 and *** when p<0.001. Results of statistical tests between the two groups of patients are indicated by symbols as follows: § when p<0.05; §§ when p<0.001 and §§§ when p<0.0001. (0.04 MB PDF) [file ppat.1000631.s003.pdf]

Table S1.

MO subsets	Surface markers	Mean ± SD positive blood MO %<br>Mean ±SD MO numbers per microliter		
		Healthy malaria-exposed individuals (n=10)	Malaria patients Group 1 (n=19)	Malaria patients Group 2 (n=57)
Classical	CD56	54.1±24.6<br>178±133	12.2±21.4 **<br>71±167 **	1.4±3.3 ***<br>10±29 ***
	mIFN-γ	49.5±26.7<br>185±138	11±22 **<br>62±165 **	0.4±0.7 ***<br>3±6 ***
	mTNF-α	8.6±17.2<br>29±39	1.1±2.3 *<br>8±17 *	0.1±0.5 ***<br>1±3 ***
	CCR2+CX3CR1+	4.4±3.9<br>17±13	11.5±7.9<br>68±58 ***	1.3±1.5 *,§§§<br>7±10 * §§§
Intermediate	CD56	1.6±2.1<br>8±11	12.5±19.3 *<br>81±138 *	19.5±21***<br>118±173 *** §
	mIFN-γ	1±1.1<br>4±4	7.9±8.8 **<br>48±61 **	11.7±14.3***<br>78±142 ***
	mTNF-α	0.6±0.5<br>3±3	3.1±4.5 *<br>16±18 **	3.6±6.7*<br>24±50 **
	CCR2+CX3CR1+	0.8±0.8<br>4±3	8.3±7.8 ***<br>55±84 ***	2.2±1.6***,§§<br>12±11 ** §
Pro-inflammatory	CD56	4.3±3.1<br>16±14	12.7±8.3 **<br>68±57 **	23.1±18.4***,§<br>136±139 ***
	mIFN-γ	3.9±2.3<br>14±9	11.8±10.1 *<br>64±67 *	19.2±19 **<br>124±154 ***
	mTNF-α	4.2±1.4<br>14±6	9.5±9.8<br>47±52 **	13.2±14.8 ***<br>81±117 ***
	CCR2+CX3CR1+	0.8±0.4<br>3±2	11±8.3 ***<br>63±55 ***	10.9±9.8 ***<br>66±78 ***
